# Fingerprinting the spatial sources of fine-grained sediment deposited in the bed of the Mehran River, southern Iran

**DOI:** 10.1038/s41598-022-07882-1

**Published:** 2022-03-10

**Authors:** Atefe Fatahi, Hamid Gholami, Yahya Esmaeilpour, Aboalhasan Fathabadi

**Affiliations:** 1 Department of Natural Resources Engineering, University of Hormozgan, Bandar-Abbas, Hormozgan Iran; 2 Department of Range and Watershed Management, Gonbad Kavous University, Gonbad Kavous, Golestan Province Iran

**Keywords:** Environmental sciences, Hydrology

## Abstract

Accurate information on the sources of suspended sediment in riverine systems is essential to target mitigation. Accordingly, we applied a generalized likelihood uncertainty estimation (GLUE) framework for quantifying contributions from three sub-basin spatial sediment sources in the Mehran River catchment draining into the Persian Gulf, Hormozgan province, southern Iran. A total of 28 sediment samples were collected from the three sub-basin sources and six from the overall outlet. 43 geochemical elements (e.g., major, trace and rare earth elements) were measured in the samples. Four different combinations of statistical tests comprising: (1) traditional range test (TRT), Kruskal–Wallis (KW) H-test and stepwise discriminant function analysis (DFA) (TRT + KW + DFA); (2) traditional range test using mean values (RTM) and two additional tests (RTM + KW + DFA); (3) TRT + KW + PCA (principle component analysis), and; 4) RTM + KW + PCA, were used to the spatial sediment source discrimination. Tracer bi-plots were used as an additional step to assess the tracers selected in the different final composite signatures for source discrimination. The predictions of spatial source contributions generated by GLUE were assessed using statistical tests and virtual sample mixtures. On this basis, TRT + KW + DFA and RTM + KW + DFA yielded the best source discrimination and the tracers in these composite signatures were shown by the biplots to be broadly conservative during transportation from source to sink. Using these final two composite signatures, the estimated mean contributions for the western, central and eastern sub-basins, respectively, ranged between 10–60% (overall mean contribution 36%), 0.3–16% (overall mean contribution 6%) and 38–77% (overall mean contribution 58%). In comparison, the final tracers selected using TRT + KW + PCA generated respective corresponding contributions of 1–42% (overall mean 20%), 0.5–30% (overall mean 12%) and 55–84% (overall mean 68%) compared with 17–69% (overall mean 41%), 0.2–12% (overall mean 5%) and 29–76% (overall mean 54%) using the final tracers selected by RTM + KW + PCA. Based on the mean absolute fit (MAF; ≥ 95% for all target sediment samples) and goodness-of-fit (GOF; ≥ 99% for all samples), GLUE with the final tracers selected using TRT + KW + PCA performed slightly better than GLUE with the final signatures selected by the three other combinations of statistical tests. Based on the virtual mixture tests, however, predictions provided by GLUE with the final tracers selected using TRT + KW + DFA and RTM + KW + DFA (mean MAE = 11% and mean RMSE = 13%) performed marginally better than GLUE with RTM + KW + PCA (mean MAE = 14% and mean RMSE = 16%) and GLUE with TRT + KW + PCA (mean MAE = 17% and mean RMSE = 19%). The estimated source proportions can help watershed engineers plan the targeting of conservation programmes for soil and water resources.

## Introduction

Accelerated soil erosion by water is an environmental threat on different continents including Asia (e.g. Iran, China and India), North America (e.g., Canada), Europe (especially the Mediterranean regions) and Africa^1–4^. Elevated suspended sediment loads in riverine systems resulting from the accelerated erosion due to human activities are a serious threat to the sustainable management of watersheds and ecosystem services therein worldwide^5^. Consequently, identifying the sources of suspended sediment in a watershed is essential to target mitigation and to help remedy problems such as eutrophication, and siltation of reservoirs. In particular, well-designed policies and control measures for protecting finite soil and water resources are dependent on reliable and scale-appropriate information on the key sources of the sediment problem which in manifested in the form of both on-site and off-site impacts^5^.

Today, sediment source fingerprinting (SSF) is increasingly applied to document sediment sources at multiple scales in differing environments. For example, SSF has been applied to quantify the provenance of riverine suspended sediment^6–9^, aeolian sands^10–18^, atmospheric dust^19–21^ and loess deposits^22^. SSF is founded on measuring the different properties of watershed source material and target sediment samples and their comparison. To date, the different properties used in SSF include colour, mineralogy, geochemical elements (e.g., major, trace and rare earth (REE) elements), isotopic signatures and ratios (e.g., ^87^Sr/^86^Sr, δ^13^C and δ^15^N), REE indices, weathering indices, fallout radionuclides (FRNs) and absolute particle size^23–28^.

In the last two decades, efforts exploring the uncertainties associated with both aeolian and fluvial SSF results has attracted increasing attention^5,7,29^. The frameworks used to quantify uncertainty associated with SSF estimates can be divided into three groups comprising Monte Carlo simulation—the most commonly applied framework^1,30,31^, Bayesian approaches^11,22,32,33^ and generalized likelihood uncertainty estimation (GLUE)^34^. Among these three frameworks, the GLUE model has been used far less frequently and, in many cases, has been applied in conjunction with quantifying the provenance of aeolian, rather than fluvial, sediments^14,20–22^. To the best of our knowledge, GLUE has not been used to quantify uncertainty associated with estimating the spatial sources of fluvial suspended sediment in river catchments. Despite this less frequent application of GLUE, it is useful to bear in mind that Bayesian modelling, as an alternative to GLUE, is more sophisticated but equally more demanding, since it uses different distributions and transformations (e.g., posterior and prior, Dirichlet), centered log ratio (CLR)^35^, additive log-ratio (ALR)^36^ and iso-metric log ratio (ILR)^37^) in the data structure. Regardless of the approach used to estimate uncertainties associated with predicted sediment source proportions, the uncertainties associated with the SSF approach may originate from a variety of sources, including within-source group tracer variability, tracer selection, limited numbers of source material or target sediment samples, laboratory analyses, and source group classification^5,28,38^.

No records of water discharge or sediment yield are available for the study area, but the mean annual runoff is estimated to be ca. 55 mm, and the specific sediment yield for the Hormozgan province is 1300 t km^−2^ year^−1^^23^. Both scientifically and managerially, fine sediment particles are an important vector for the transfer, dispersal and fate of nutrients and contaminants, whilst also causing detrimental impacts on all aquatic trophic levels including diatoms, macroinvertebrates, macrophytes and fish^5^. Given the above background, the primary goal of this study was to apply geochemical SSF within GLUE framework in the estimation of sub-basin spatial sediment source contributions in the arid Mehran River catchment in southern Iran, which drains into the Persian Gulf. The accuracy of GLUE predictions generated using four different sets of statistical tests for discriminating three sub-basin spatial sources was evaluated using 10 virtual sediment (VS) samples with known source contributions using the root mean square error (RMSE) and mean absolute error (MAE).

## Materials and methods

### Study area

**Figure 1:**
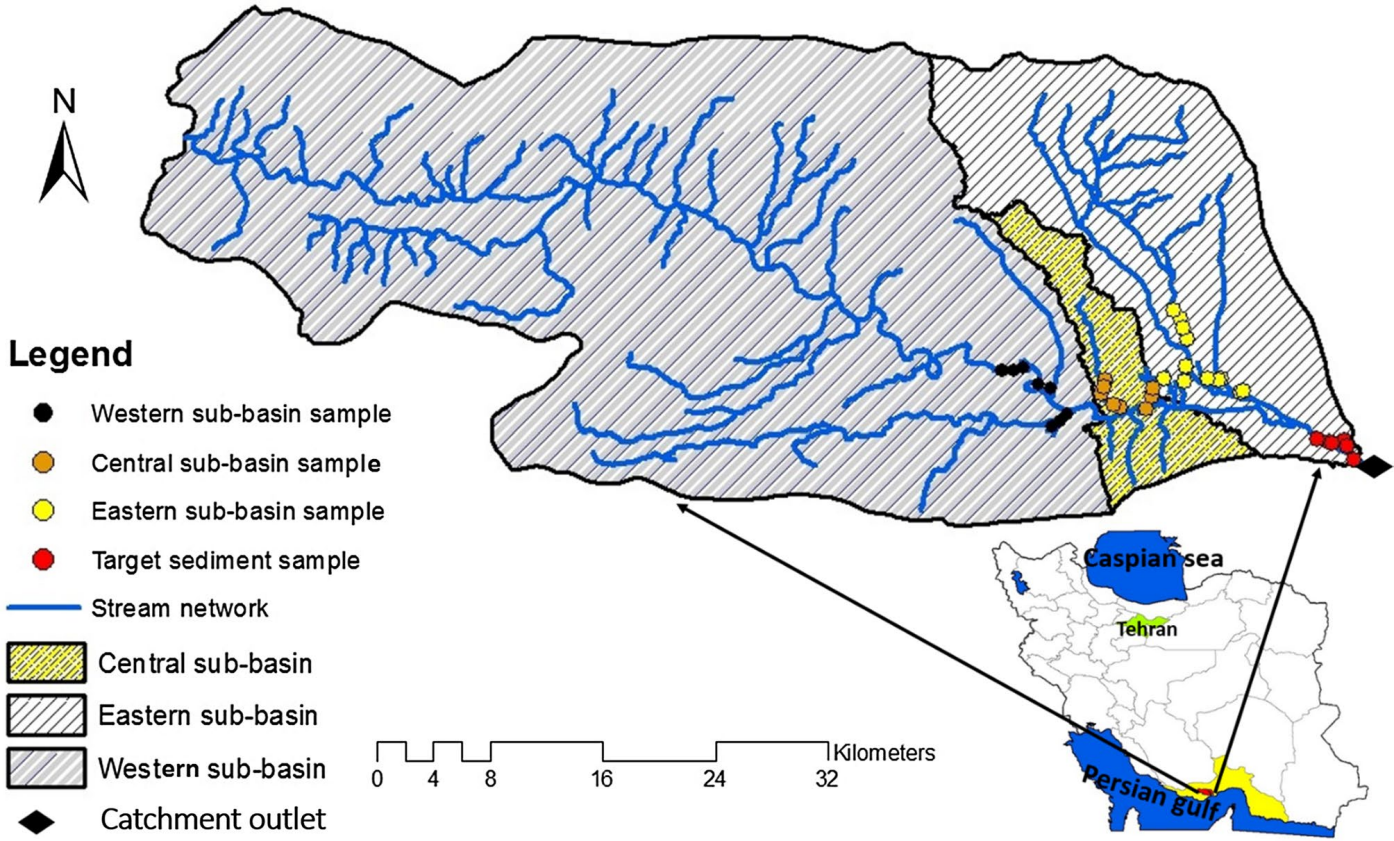
Location of the sub-basin and catchment outlet sediment sampling sites in the study area in Hormozgan province, Iran. The red and yellow shading in the bottom map indicate the study catchment and Hormozgan province, respectively. This map was generated in ArcGIS 10.4.1 (https://www.esri.com/en-us/about/about-esri/overview).

The Mehran River catchment (2142 km^2^) with mean annual rainfall 140 mm is located in the western part of Hormozgan province, southern Iran (26° 42′ to 27° 16′ N, and 54° 30′ to 55° 26′ ″) (Fig. 1). The study area is surrounded by Bandar-e-Khamir and Bandar-e-Langeh on the eastern and western sides, respectively. It can be divided into three sub-basins comprising a western sub-basin (1611 km^2^), a central sub-basin (106 km^2^) and an eastern sub-basin (425 km^2^). The river is 86 km long. Elevation ranges between − 44 m in the eastern part of the study area in the vicinity of the catchment outlet to 1857 m in the northwestern part. Slopes range between 0 to 79%. Due to existing mangrove forests in the vicinity of the outlet located on the northern coast of the Persian Gulf, the study catchment is earmarked as being important environmentally. Geologically, the study area is underlain by diverse geological units including low level piedment fan and valley terrace deposits, Jahrum formation (including grey and brown weathered, massive dolomite, low weathered thin to medium-beded dolomite and massive, feature forming, and buff dolomitic limestone), Gachsaran formation (including anhydrite, salt, grey and red marl alternating with anhydrite, argillaceous limestone and limestone), undivided Asmari and Jahrum formation, Mishan formation (or low weathering gray marls alternating with bands of more resitant shelly limestone), Karaj formation (including well bedded green tuff and tuffaceous shale), Bakhtyari formation (or alternating hard of consolidated, massive, feature forming conglomerate and low-weathering cross-bedded sandstone), undivided Bangestan group, mainly limestone and shale, Albian to Companian, comprising the following formations: Kazhdumi, Sarvak, Surgah and Ilam, Aghajari formation (including brown to grey, calcareous, feature-forming sandstone and low weathering, gypsum- veined, red marl and siltstone), and Razak formation (red, grey, and green silty marls inter-bedded with subordinate silty limestone and minor sandstone ribs). The main channel of the Mehran River is established on Quaternary fans and terraces.

### Sampling, sample preparation and laboratory analysis

**Figure 2:**
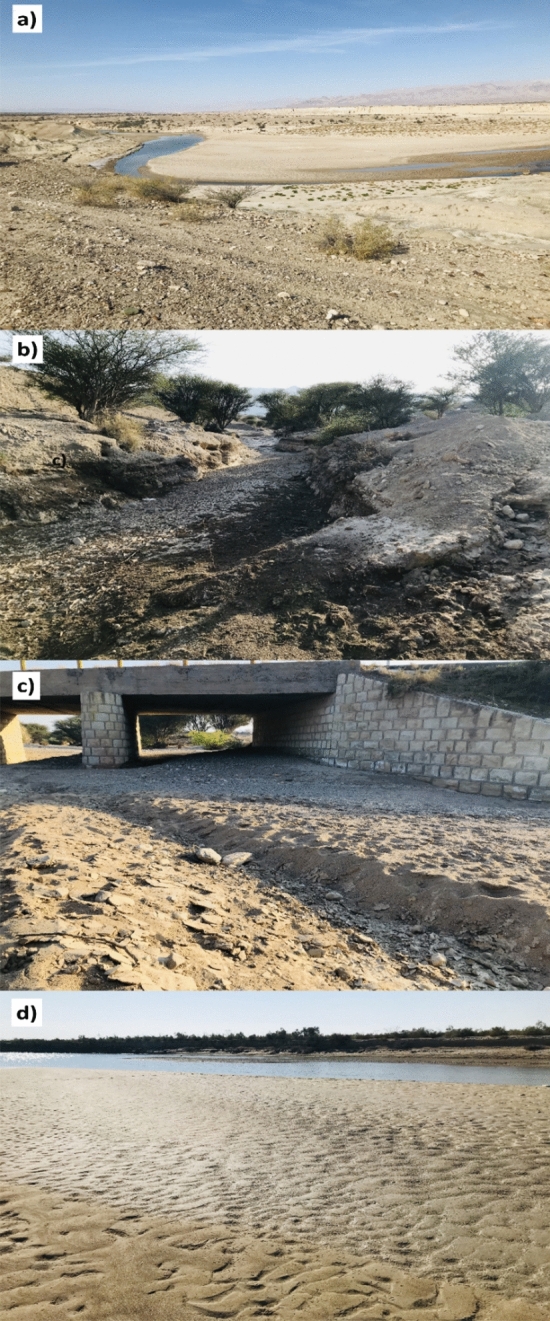
Photographs showing the sediment deposits where the samples were collected from: (**a**) western sub-basin; (**b**) central sub-basin; (**c**) eastern sub-basin, and; (**d**) the main channel of the Mehran River in the vicinity of the overall catchment outlet.

**Figure 3:**
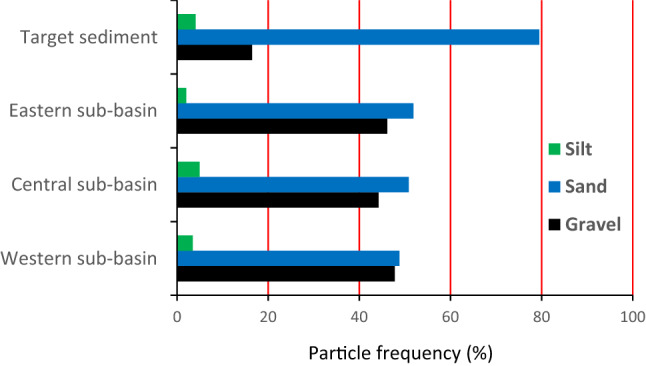
The mean of the particles size for three sources and target sediment.

Based on the sub-basin map (Fig. 1), potential spatial sediment sources in the Mehran River catchment were classified as the western, central and eastern sub-basins. A total of 28 surficial samples were collected from the fine-grained materials deposited in the bed of the main channel of the sub-basin spatial sources, comprising eight for the western, eight for the central and 12 for the eastern sub-basin, respectively (Fig. 2a–c). A total of six target sediment samples were collected from the fine-grained sediments deposited in the bed of the Mehran River main stem in the vicinity of the overall outlet (Fig. 2d). Samples were air dried and sieved to separate the < 63 μm fraction. The mean of the particles size for three sources and target sediment samples is presented in Fig. 3. The conventional < 63 μm fraction was selected as this is the most geochemically active^39^. Aqua regia was used to digest the sieved samples and then the solutions were analysed for tracer pseudo-content using inductively coupled plasma atomic emission spectroscopy (ICP-OES) in the Central Laboratory of the University of Hormozgan. In total, 43 geochemical elements (Al, As, Ba, Be, Bi, Cu Ce, Co, Cr, Cs, Er, Fe, K, Li, Mo, Ga, Gd, Hf, Ho, In, Mg, Mn, Na, Nb, Ni,Te, Zn, P, Pb, S, Sc, Sm, Sn, Ta, Tb, V, W, Ag, Zr, Ca, Eu and La) were measured in the 34 source material and target sediment samples.

### Discriminating the spatial sources of the target sediment samples

A wide range of methods including individual statistical tests such as the Kruskal–Wallis H test, stepwise discriminant function analysis, principle component analysis, or combined statistical procedures, are used to discriminate the potential sources of target sediment samples^29^. Here, we used four combinations of statistical procedures. The first comprised a TRT (based on the minimum and maximum ranges of tracer concentrations in the source and target sediment samples) for tracer conservation plus the (KW test for individual tracer discriminatory power and DFA for composite signature discriminatory power. The second comprised a RTM (range test using mean tracer values in source and target sediment samples) in combination with KW and DFA. The third combination of tests comprised TRT, KW and PCA. The fourth combination combined RTM, KW and PCA. Tracer bi-plots was used for further assessment of source discrimination provided by each final composite signature and of tracer conservation.

### Generalised likelihood uncertainty estimation (GULE)

GLUE was first applied to uncertainty modelling and sensitivity analysis for hydrological models by^40^. In more recent years (2019 to present) GLUE modelling has been applied to quantify uncertainty associated with source contributions to sampled loess deposits, and aeolian dust and sands in Central Asia, Iran and Australia^14,20–22^. Based on GLUE, we can quantify the uncertainties associated with SSF results via the following five key steps:

*Step 1* Application of LHS (Latin Hypercube Sampling) for the random sampling of tracer parameter sets (10,000 iterations). At this stage, two boundary constraints (0 ≤ $x_{j}$ ≤ 1; and $\sum x_{j} = 1$) must be satisfied^30^;

*Step 2* Use of the Nash–Sutcliffe coefficient (NSC) as the likelihood function^21^:

1 $$ NSC = 1 - \frac{{\mathop \sum \nolimits_{i = 1}^{n} \left( {a_{o} - a_{s} } \right)^{2} }}{{\mathop \sum \nolimits_{i = 1}^{n} \left( {a_{o} - am_{o} } \right)^{2} }} $$

where *a*_*s*_ and *a*_*o*_ indicate the simulated *i*th final tracer concentration and the measured *i*th final tracer concentration, respectively. *am*_*o*_ represents the mean value of the *i*th measured tracer concentration in the target sediment sample;

*Step 3* Application of an un-mixing model as follows:

2 $$ A_{ts} = B_{s} \times C $$

where *C* is an m dimensional column vector of the spatial source contributions, $A_{ts}$ indicates an n-dimensional column vector of tracer concentrations in the target sediment sample, and $B_{s}$ represents an n × m dimensional matrix representing the mean tracer concentrations in the spatial sub-basin sources;

*Step 4* Division of the tracer parameter sets into behavioural and non-behavioural types, and;

*Step 5* Re-scaling of the likelihood weights for the behavioural parameter sets.

More details for GLUE modelling can be found in^14,21^.

### Assessment of GLUE performance

Two statistical measures (mean absolute fit—MAF and goodness-of-fit—GOF)^20,41–43^ were applied to assess the performance of GLUE in estimating the measured tracer concentrations in the target sediment samples collected from the outlet of the Mehran River catchment, viz.:

3 $$ MAF = 1 - \frac{1}{n} \times \left( {\mathop \sum \limits_{i = 1}^{n} \left| {\frac{{b_{i} - \mathop \sum \nolimits_{j = 1}^{n} x_{j} a_{j.i} }}{{b_{i} }}} \right|} \right) $$

4 $$ GOF = 1 - \frac{1}{n} \times \mathop \sum \limits_{i = 1}^{n} \left\{ {\left( {\frac{{\left| {b_{i} - \mathop \sum \nolimits_{i = 1}^{m} x_{j} a_{j,i} } \right|}}{{b_{i} }}} \right)^{2} } \right\} $$

where n and m are the number of tracers in the final composite signature and number of sub-basin spatial sources (m = 3), respectively. $b_{i}$ is the concentration of the final tracer (i) measured in the target sediment sample, ${ }x_{j}$ indicates the relative contribution of source (j) to the target sediment sample, and $a_{j,i} { }$ represents the mean concentration of the final tracer (i) in the sub-basin spatial source (j).

The accuracy of the GLUE predictions of spatial source contributions was assessed using 10 VS mixtures^20,22,31^. Here, the root mean square error (RMSE) and mean absolute error (MAE) were used to compare the GLUE predictions with known source contributions from the three sub-basin spatial sources, viz.:

5 $$ RMSE = \sqrt {\frac{{\mathop \sum \nolimits_{i = 1}^{n} (X_{K} - X_{P} )^{2} }}{n}} $$

6 $$ MAE = \frac{{\mathop \sum \nolimits_{i = 1}^{n} \left| {X_{K} - X_{P} } \right|}}{n} $$

where *X*_*K*_ and *X*_*P*_ indicate the known contribution from the sub-basin spatial sources in the VS and the corresponding contribution predicted by the GLUE model, respectively. n is the number (n = 3) of sub-basin spatial sources.

**Figure 4:**
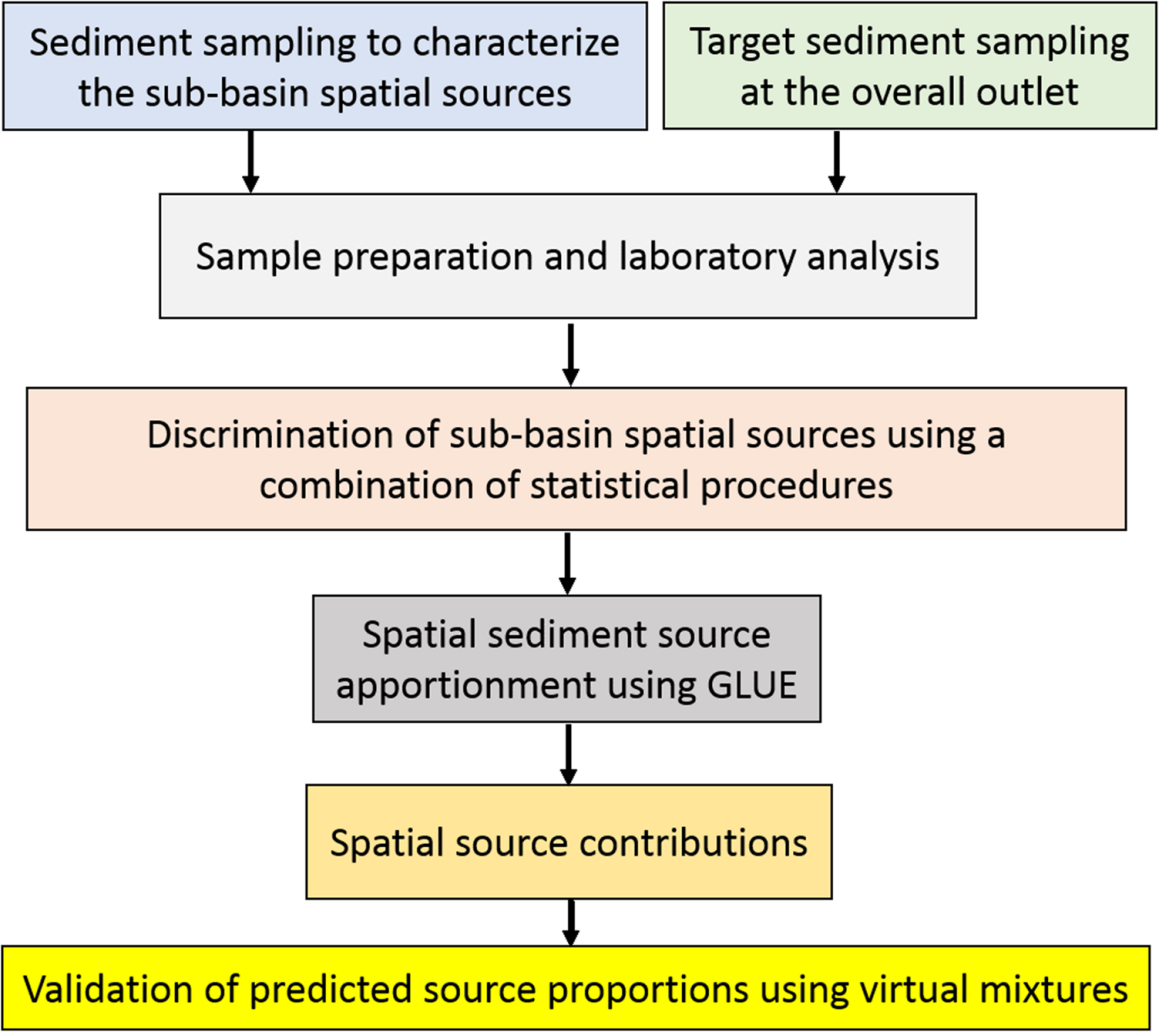
Flowchart for the GLUE methodology applied for source fingerprinting of the target sediment samples collected from the Mehran River.

A methodological flowchart is presented in Fig. 4.

## Results and discussion

### Discriminating conservative from non-conservative tracers

The selection of a combination of final geochemical tracers in a so-called composite signature for source apportionment is one of the key stages in successful SSF^29,44,45^. Based on TRT, 11 geochemical tracers (Be, Bi, Cs, Er, Fe, K, Li, Mo, Te, Zn and Cu) were identified as non-conservative, meaning that 33 geochemical elements ( Al, As, Ba, Ce, Co, Cr, Ga, Gd, Hf, Ho, Mg, Mn, Na, Nb, Ni, P, Pb, Pr, Rb, S, Sc, Sm, Sn, Ta, Tb, V, W, Ag, Zr, Ca, Eu and La) were conservative. By comparison, using RTM, 18 geochemical tracers (Al, As, Ba, Be, Co, Cr, Fe, Ga, K, Mo, Na, Pb, Sm, Sn, Ta, Tb, Te and Zn) failed the test. Logically, the results for TRT and TRM make sense since TRM is a stricter test meaning that more tracers typically fail this mathematical test for conservative behaviour. Due to immobility and low solubility, rare earth elements (REEs) (e.g., Ce, Gd, Ho, Sc, Sm, Tb, Eu and La) may be useful tracers for identifying the provenance of sediments and the formation mechanism of rocks^23,46^.Tracers failing either range test were excluded from further statistical analysis.

### Spatial sediment source discrimination

#### Stepwise DFA

The results of KW suggested that, among the 32 geochemical tracers passing the TRT, 12 (Al, Ce, Hf, Mn, Nb, P, Rb, Sc, Zr, Ca, Eu and La) were significant (*p* ≤ 0.05), whist 20 geochemical elements (As, Ba, Co, Cr, Ga, Gd, Ho, Mg, Na, Ni, Pb, Pr, S, Sm, Sn, Ta, Tb, V, W and Ag) were not statistically significant (*p* ≥ 0.05). The 12 statistically significant tracers were used in stepwise DFA for identifying the final composite signature for discriminating the sub-basin spatial sources of the target sediment samples. Based on the stepwise DFA (Table 1), three geochemical tracers (Zr, Mn and P) were selected. The final tracers were selected based on minimizing the Wilks’ Lambda values. The values of Wilks’ Lambda ranged between 13.9 (for the first step with Zr as the first tracer entered into the model) and 11.3 (for the third step with P as the third tracer entered into the model).

**Table 1:** The final composite signature selected by both TRT + KW + DFA and TRM + KW + DFA for discriminating the three sub-basin spatial sources of the six target sediment samples collected at the outlet of the study area.

Step	Final tracers	Wilks’ Lambda	
		Statistic	Sig
1	Zr	13.9	0.000
2	Mn	11.7	0.000
3	P	11.3	0.000

**Figure 5:**
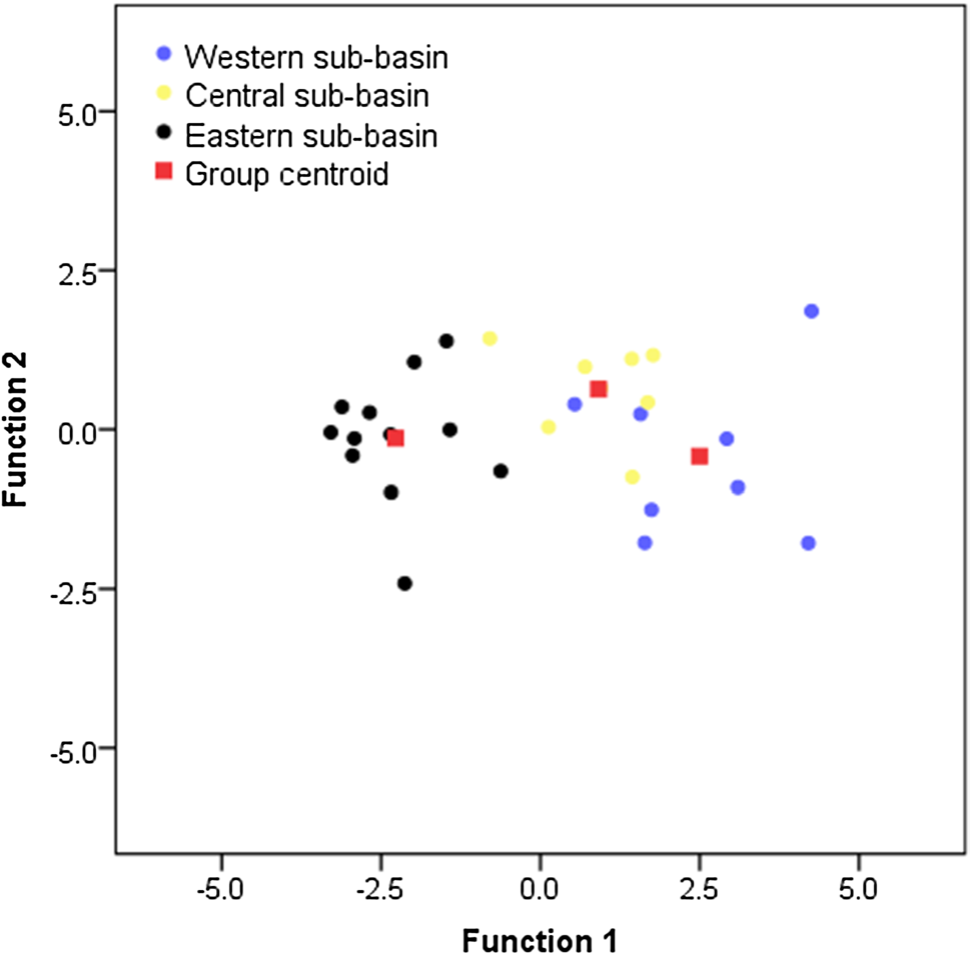
Two-dimensional scatterplot constructed based on the first and second functions of the stepwise DFA for the tracers selected using either TRT + KW + DFA or TRM + KW + DFA.

Among the 25 geochemical tracers (Bi, Ce, Cs, Er, Gd, Hf, Ho, Li, Mg, Mn, Nb, Ni, P, Pr, Rb, S, Sc, V, W, Ag, Zr, Ca, Cu, Eu and La) passing RTM, 12 geochemical proprieties (Bi, Cs, Er, Gd, Ho, Mg, Ni, Pr, S, V, W, and Ag) were not statistically significant (with *p* ≥ 0.05) according to KW, whereas 13 (Ce, Hf, Li, Mn, Nb, P, Rb, Sc, Zr, Ca, Cu, Eu and La) were significant (*p* ≤ 0.05). Overall, the results of stepwise DFA for tracers passing RTM + KW were the same as those identified using TRT + KW + DFA (Table 1, Fig. 5).

The results of the stepwise DFA are summarised in Fig. 5 and Table 2. Based on these results, a combination of Zr, Mn and P were able to correctly classify 89.3% of the sub-basin spatial source sediment samples (Fig. 5).

**Table 2:** Classification results for the three sub-basin spatial sediment sources using stepwise DFA.

No	Predicted group membership			Total
	Western sub-basin	Central sub-basin	Eastern sub-basin	
**Count**				
Western sub-basin	6	2	0	8
Central sub-basin	1	7	0	8
Eastern sub-basin	0	0	12	12
**Percent**				
Western sub-basin	75	25	0	100
Central sub-basin	12.5	87.5	0	100
Eastern sub-basin	0	0	100	100

#### Principle component analysis (PCA)

**Figure 6:**
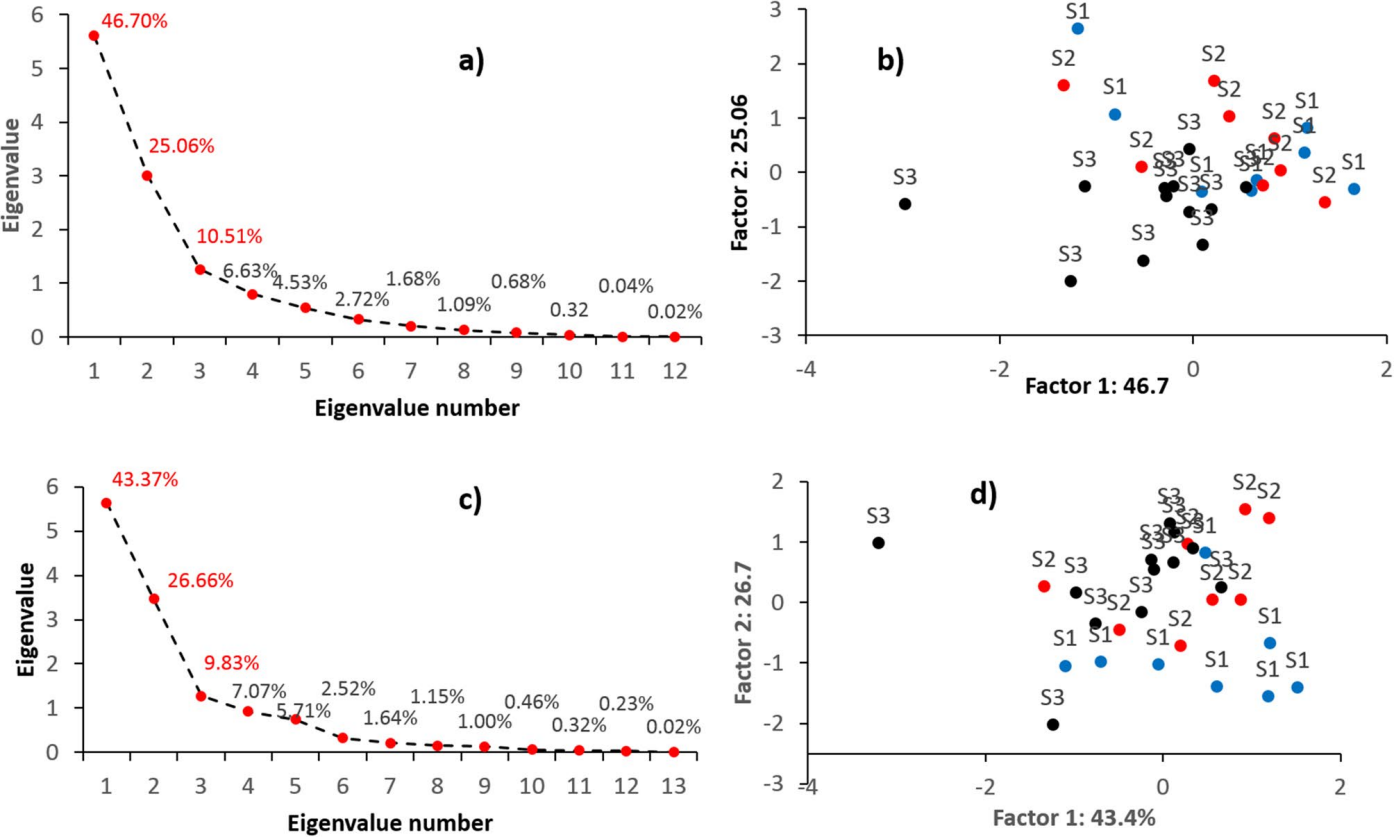
(**a**) Scree plot output from the PCA for the spatial sediment source discrimination based on geochemical tracers selected by TRT + KW, (**b**) projection of the cases on the principal component plane using PCA based on geochemical tracers selected by TRT + KW, (**c**) Scree plot output from the PCA for the spatial sediment source discrimination based on geochemical tracers selected by RTM + KW, and; (**d**) projection of the cases on the principal component plane using PCA based on geochemical tracers selected by RTM + KW. S1, S2 and S3 indicate samples collected from the spatial sources represented by the western, central and eastern sub-basins, respectively.

12 (Al, Ce, Hf, Mn, Nb, P, Rb, Sc, Zr, Ca, Eu and La) and 13 (Ce, Hf, Li, Mn, Nb, P, Rb, Sc, Zr, Ca, Cu, Eu and La) geochemical tracers passing TRT + KW and RTM + KW (Table 3), respectively, were entered into the PCA. Based on Table 3 and Fig. 6, the first three PC yielded the most interpretable factor pattern. The initial eigenvalues for components 1 to 3 ranged between 5.6 and 1.26 (Fig. 6a). The percentage of the variance explained by these components was calculated as 30.7%, 25.9% and 25.6%, respectively. Based on Fig. 6c, the variance of PC1, PC2and PC3 was 29.3%, 26.6% and 23.9%, respectively, whereas the initial eigenvalue for these components was estimated to be 5.63, 3.46 and 1.28, respectively. Based on Fig. 6b,d, projection of the sample cases on the PC plane using PCA indicates that the final set of tracers, selected using a combination of either TRT + KW + PCA or TRM + KW + PCA, provided relatively good discrimination between the three sub-basin spatial sediment sources.

**Table 3:** Results of PCA based on tracers passing TRT + KW and RTM + KW.

Tracer	PC1	PC2	PC3
**TRT + KW + PCA**			
Al	0.05	− 0.02	0.97
Ce	0.94	0.26	0.03
Hf	0.23	0.75	0.01
Mn	0.05	0.11	0.84
Nb	0.29	0.71	0.30
P	0.74	0.46	0.05
Rb	− 0.16	− 0.52	0.73
Sc	0.12	0.38	0.86
Zr	0.26	0.86	0.13
Ca	0.48	0.69	− 0.19
Eu	0.91	0.28	0.12
La	0.95	0.21	0.01
**RTM + KW + PCA**			
Ce	0.94	− 0.00	0.26
Hf	0.23	− 0.00	0.76
Li	− 0.28	0.88	− 0.07
Mn	0.11	0.87	0.15
Nb	0.28	0.19	0.76
P	0.73	− 0.03	0.47
Rb	− 0.21	0.72	− 0.40
Sc	0.13	0.82	0.45
Zr	0.26	0.03	0.88
Ca	0.53	− 0.22	0.63
Cu	0.15	0.80	− 0.05
Eu	0.92	0.01	0.27
La	0.94	− 0.01	0.21

### Bi-plots as a further test for identify conservative tracers

**Figure 7:**
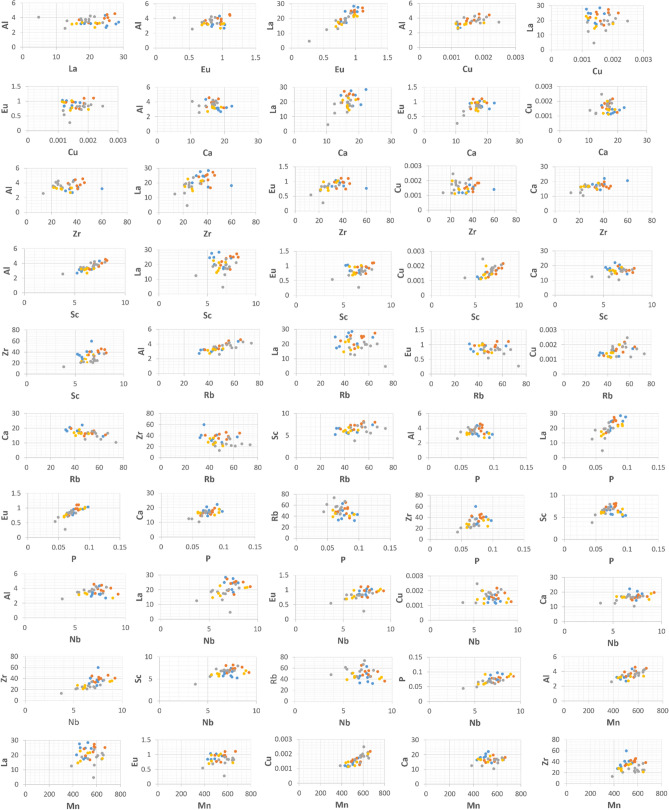

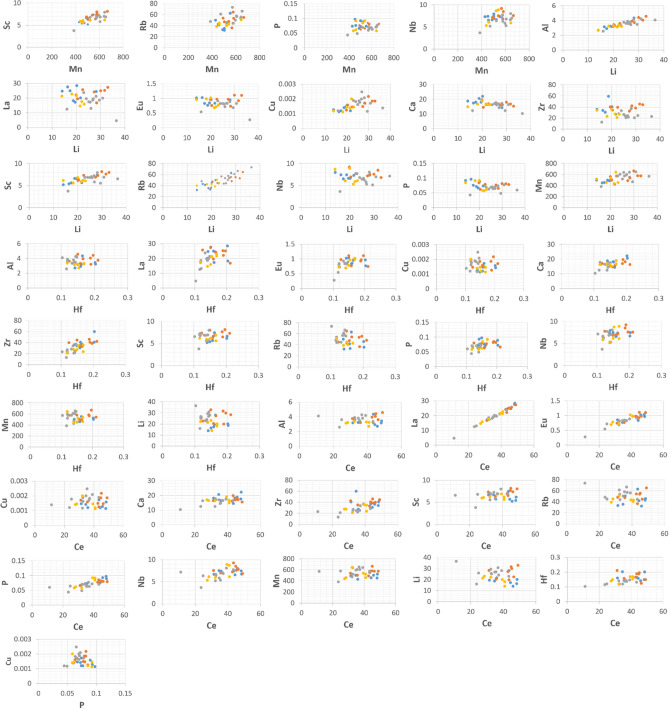
The bi-plots of the geochemical tracers comprising the final composite signatures selected using TRT + KW + DFA, RTM + KW + DFA, TRT + DFA + PCA and RTM + DFA + PCA for discriminating the three sub-basin spatial sediment sources. The blue, orange, grey and gold points indicate the samples collected from the western, central and eastern sub-basins and the target sediment samples, respectively.

Bi-plots of the 14 geochemical tracers (Al, Ce, Hf, Li, Nb, Rb, Sc, Ca, Cu, Eu, La, Zr, Mn and P) comprising the final composite signatures selected using TRT + KW + DFA, RTM + KW + DFA, TRT + KW + PCA and RTM + KW + PCA were constructed as a further test for geochemical tracer conservation (Fig. 7). These bi-plots confirmed the conservative behaviour of the final geochemical tracers during sediment mobilization and delivery to the sampling points at the outlet of the study catchment since the sub-basin spatial source and target sediment samples plotted in the same space on each plot. Plots wherein the samples do not fall in the same space indicate non-conservative behaviour of the geochemical tracers in question. Several studies have reported the inclusion of bi-plots for identifying conservative tracers in SSF^28,47–49^.

### The uncertainty ranges of the source contributions and their cumulative distributions estimated by GLUE

**Figure 8:**
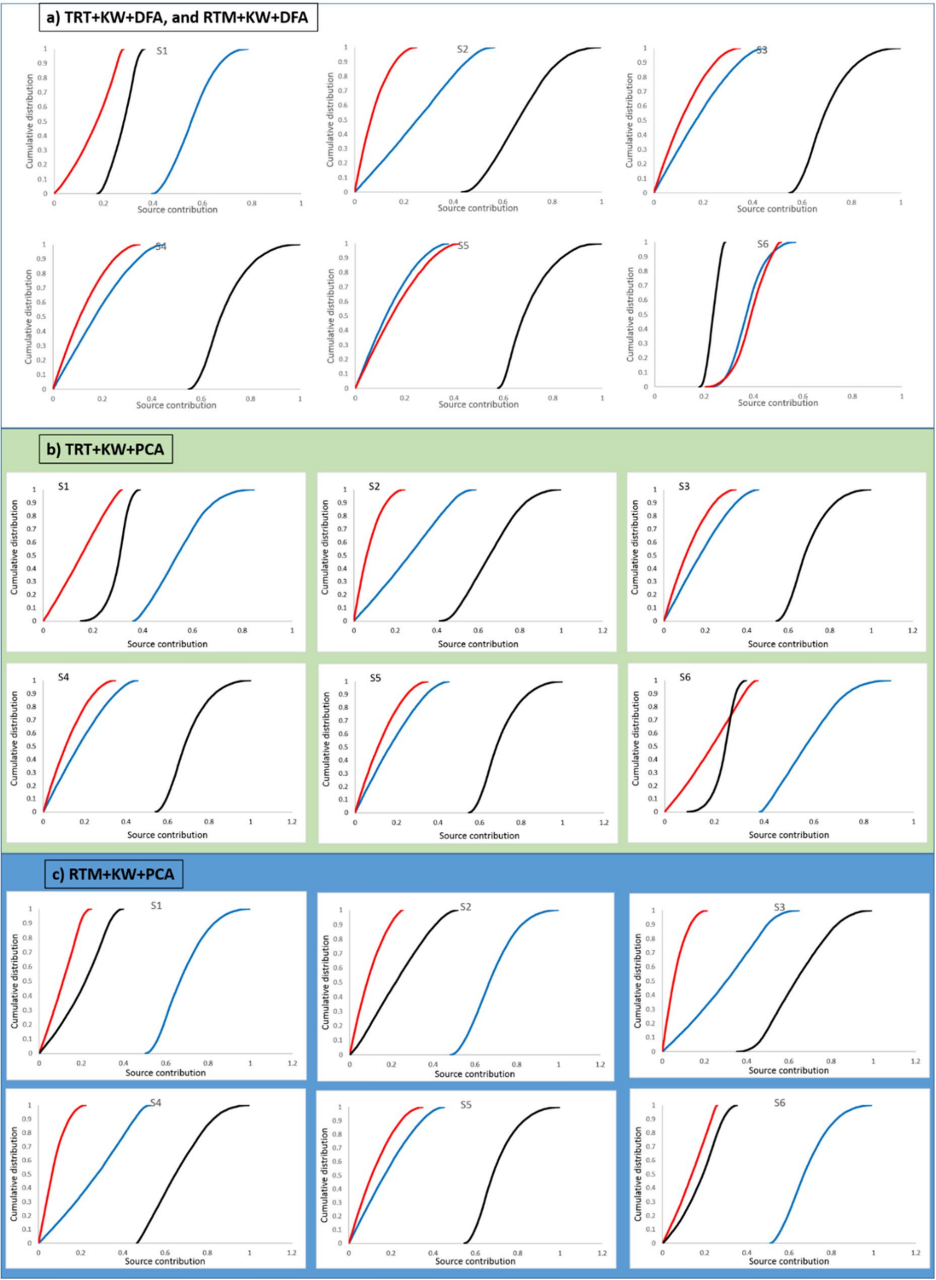
The cumulative distributions for the predicted contributions from the three sub-basin spatial sources (western sub-basin—blue line; central sub-basin—red line, and; eastern sub-basin—black line) modelled using GLUE based on the final composite signatures selected using the four different combinations of statistical tests.

The uncertainty ranges (with 95% confidence limits) of the estimated source contributions and the their cumulative distributions for the six target sediment samples collected from the outlet of the Mehran River estimated by GLUE using the signatures selected by the different combinations of the statistical tests are presented in Fig. 8. Using the final composite signature selected by TRT + KW + DFA and RTM + KW + DFA (Fig. 8a), the contributions from the western sub-basin to target sediment sample S1 were predicted to dominate and ranged between 42 and 72%, compared with 1 to 27% from the central sub-basin, and 19 to 35% from the eastern sub-basin. For target sediment sample S2, the corresponding respective contributions, using the same final composite signature, ranged between 1–51%, 0.2–21% and 48–90%. The western sub-basin contributed 1–40% for S3, whereas the central and eastern sub-basins contributed 0.5 to 30%, and 57 to 90%, respectively. The ranges of the contributions from the western, central and eastern sub-basins spatial sources to S4 ranged between 0.9 to 41%, 5 to 30%, and 57 to 91%, respectively. The predicted contributions from the western sub-basin to S5, ranged between 0.6–33%, whereas the corresponding contributions from the central and eastern sub-basins ranged between 0.7–37% and 59–90%, respectively. The predicted contributions from the western sub-basin to S6 ranged between 26 to 51%, whereas the contributions from the central and eastern sub-basins ranged between 26 to 49%, and 19 to 28%, respectively.

The results for the predicted spatial source contributions using GLUE and the final composite signature selected by TRT + KW + PCA (Fig. 8b) suggested that the ranges of the contributions from the western, central and eastern sub-basins spatial sources to S1 were 38 to 76%, 1 to 30%, and 20 to 37%, respectively. The western sub-basin contributed 1–52% of S2, whereas the central and eastern sub-basins contributed 0.2 to 20%, and 46 to 91%, respectively. The corresponding respective contributions to S3 ranged between 1–41%, 0.5–39% and 57%-91%. For target sediment sample S4, the contribution from the western sub-basin ranged between 0.8 and 41%, whereas the corresponding contributions from the central and eastern sub-basins ranged between 0.5–30% and 57–91%, respectively. The ranges of the contributions from the western, central and eastern sub-basins spatial sources to S5 were 0.8 to 40%, 5 to 30%, and 57 to 91%, respectively. The predicted contributions from the western, central and eastern sub-basins to S6, ranged between 40–81%, 1–35% and 14–31%, respectively.

Finally, the results for the predicted spatial source apportionment using GLUE and the final composite signature selected by RTM + KW + PCA (Fig. 8c), indicated that the contribution of the western sub-basin to target sediment sample S1, ranged between 53–91%, whereas the corresponding contributions from the central and eastern sub-basins range between 0.6–22% and 1–37%, respectively. The contributions from the western sub-basin to target sediment sample S2 were predicted to dominate and ranged between 51 and 90%, compared with 0.3 to 23% from the central sub-basin, and 15 to 47% from the eastern sub-basin. The eastern sub-basin was predicted to be the dominant source of target sediment samples S3 (42–91%), S4 (48–90%) and S5 (57–91%), whereas the corresponding predicted contributions from the western and central spatial sub-basins ranged between 2–56%, 2–49% and 1–40%, and 0.3–17%, 3–19% and 0.4–30%, respectively. The ranges of the contributions from the western, central and eastern sub-basins spatial sources to S6 were 53 to 90%, 7 to 25%, and 2 to 32%, respectively.

### Assessment of the GLUE predictions using MAF and GOF estimators

**Figure 9:**
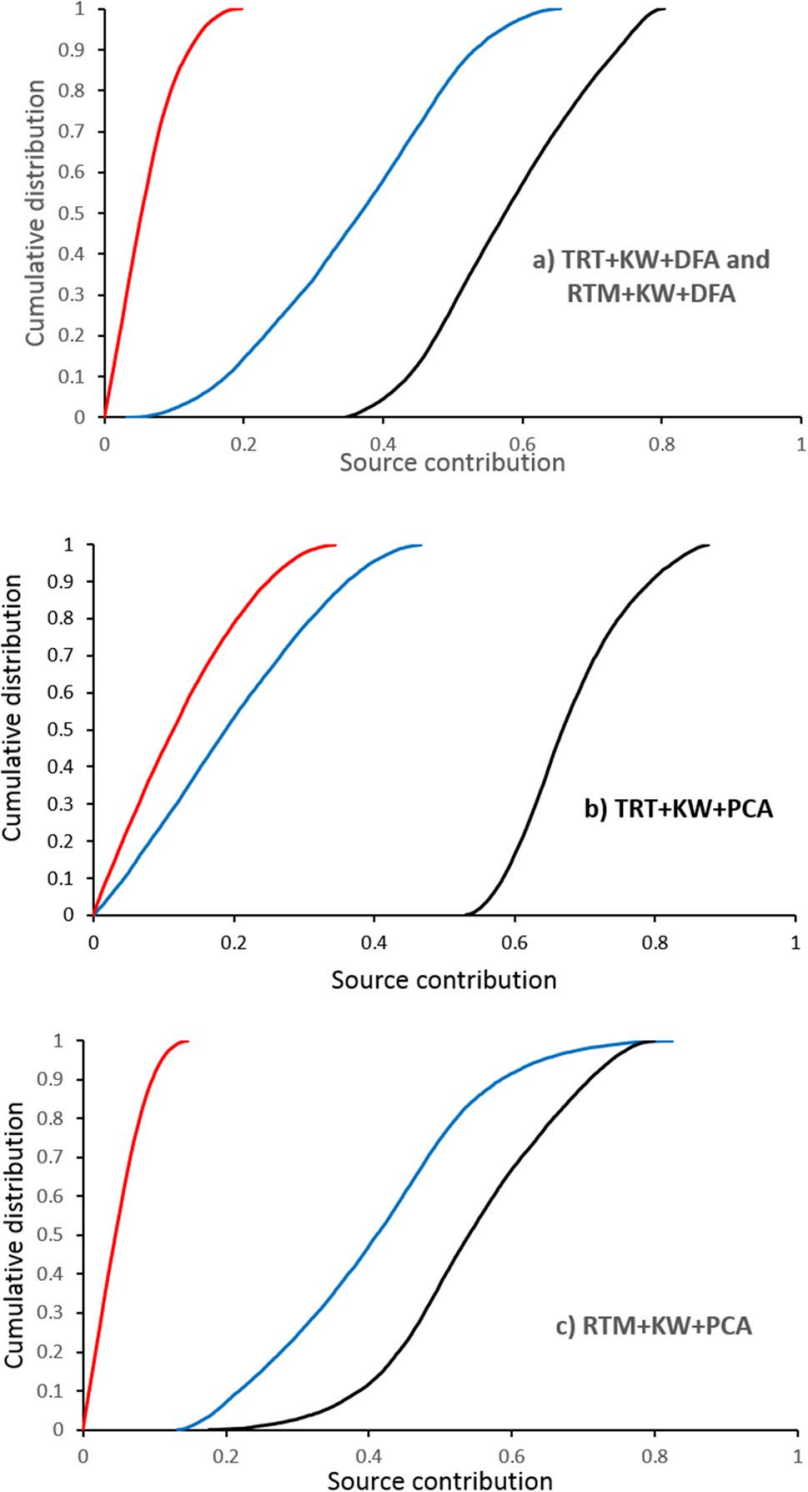
The cumulative distributions for the overall mean contributions of the three sub-basin spatial sediment sources (western sub-basin—blue line, central sub-basin—red line and eastern sub-basin—black line) modelled using GLUE with the different combinations of the statistical tests.

The contributions from the three sub-basins estimated by GLUE (with 95% confidence limits) using the four different statistical combinations (TRT + KW + DFA, RTM + KW + DFA, TRT + KW + PCA and RTM + KW + PCA) including their overall means and the corresponding estimates of MAF and GOF are presented in Fig. 9 and Table 4. The results of apportionment by GLUE using TRT + KW + DFA and RTM + KW + DFA shows that the overall mean contributions from the western, central and eastern sub-basins ranged between 10–60%, 0.3 to 16% and 38% to 77%, respectively (Fig. 9a), and the overall respective mean contributions were estimated at 36%, 6% and 58% (Table 4). Based on the MAF (≥ 80%, exception MAF = 78% for target sediment sample S2) and GOF (≥ 95%) results, the GLUE procedure was able to predict the measured concentrations in the target sediment samples of the tracers comprising these two final composite signatures. Based on the final composite signature selected using TRT + KW + PCA, the range of the overall mean contributions of the western sub-basin was predicted to be 1–42% (with an overall mean of 20%), whereas the corresponding contributions of the central and western sub-basin spatial sources ranged between 0.5–30% (overall mean 14%) and 55–84% (overall mean 68%), respectively (Fig. 9b). Based on MAF (> 84%) and GOF (≥ 97%), the predicted tracer values using GLUE in combination with the final composite signature selected by TRT + KW + PCA were more accurate than the predictions provided by GLUE with TRT + KW + DFA, RTM + KW + DFA and RTM + KW + PCA. The overall mean contributions, provided by GLUE in combination with the final composite signature selected using RTM + KW + PCA, from the western, central and eastern sub-basin spatial sources ranged between 17–69% (overall mean 41%), 0.2–12% (overall mean 5%) and 29–76% (overall mean 54%), respectively (Fig. 9c). Among the four different statistical combinations, predictions provided by GLUE with RTM + KW + PCA, with a MAF = 89% and a GOF = 99%, had the lowest accuracy with respect to predicting the measured tracer concentrations in the target sediment samples.

**Table 4:** The contributions of the three sub-basin spatial sources estimated by GLUE and the final composite signatures selected using different statistical tests and the corresponding values of MAF and GOF for predicting the measured geochemical tracer concentrations in the six target sediment samples (S1-S6).

Statistical tests combinations	Target sediment sample	Western sub-basin	Central sub-basin	Eastern sub-basin	MAF (%)	GOF (%)
TRT + KW + DFA and RTM + KW + DFA	S1	56	16	28	95	100
	S2	25	8	68	78	95
	S3	18	12	70	88	99
	S4	18	12	70	92	99
	S5	14	16	70	81	96
	S6	37	39	24	93	99
	Overall mean	36	6	58	94	100
TRT + KW + PCA	S1	55	15	30	90	97
	S2	27	7	66	84	97
	S3	18	12	70	88	98
	S4	18	12	70	92	99
	S5	18	12	70	88	97
	S6	57	19	24	98	100
	Overall mean	20	12	68	95	100
RTM + KW + PCA	S1	68	11	21	79	91
	S2	68	9	23	76	92
	S3	30	6	64	84	97
	S4	27	7	66	89	98
	S5	18	12	70	84	97
	S6	68	14	18	96	100
	Overall mean	41	5	54	89	99

### Testing the predicted source proportions with virtual sediment (VS) mixtures

The ten VS samples with different known mixtures presented in Table 5 were used to evaluate the accuracy of the GLUE models using final composite signatures selected by different combinations of the statistical tests for discriminating the three sub-basin spatial sources. The values of the MAE for the GLUE with TRT + KW + DFA and RTM + KW + DFA ranged between 30% for VS7 to 2% for VS9 and VS10, whereas the corresponding values of the RMSE ranged between 2% for VS9 and 36% for VS7. The highest values for MAE and RMSE, or the lowest accuracy, was estimated for VS7 with known contributions of 60% (western sub-basin), 20% (central sub-basin) and 20% (eastern sub-basin), compared with predicted corresponding contributions of 16% (western sub-basin), 61% (central sub-basin) and 23% (eastern sub-basin). Among the VS mixtures, the highest accuracy was returned for VS9 with known contributions of 20% (western sub-basin), 20% (central sub-basin) and 60% (eastern sub-basin), and predicted contributions of 20% (western sub-basin), 17% (central sub-basin) and 63% (eastern sub-basin). The values of the MAE and RMSE were estimated to be less than 20% for eight VS mixtures (VS2, VS3, VS4, VS5, VS6, VS8, VS9 and VS10) but ≥ 28% for VS1 and VS7.

**Table 5:** Comparison of the modelled and known contributions from the three spatial sub-basin sources to the target sediment samples (VS1-VS10) using GLUE and virtual sediment (VS) mixtures.

Statistical tests combinations	VS sample no	VS mixture known source proportions			GLUE predicted source proportions			MAE	RMSE
		Western sub-basin	Central sub-basin	Eastern sub-basin	Western sub-basin	Central sub-basin	Eastern sub-basin		
TRT + KW + DFA and RTM + KW + DFA	VS1	80	10	10	43	52	5	28	33
	VS2	10	80	10	33	60	7	16	18
	VS3	10	10	80	13	17	70	6	8
	VS4	40	30	30	51	27	22	7	8
	VS5	30	40	30	32	43	25	4	4
	VS6	30	30	40	20	48	32	12	13
	VS7	60	20	20	16	61	23	30	36
	VS8	20	60	20	26	51	23	6	7
	VS9	20	20	60	20	17	63	2	2
	VS10	33.3	33.3	33.3	32	31	37	2	3
TRT + KW + PCA	VS1	80	10	10	30	66	4	38	44
	VS2	10	80	10	39	47	14	22	26
	VS3	10	10	80	14	11	75	4	5
	VS4	40	30	30	20	71	9	28	29
	VS5	30	40	30	29	21	50	14	17
	VS6	30	30	40	7	44	49	15	17
	VS7	60	20	20	62	11	27	6	7
	VS8	20	60	20	25	69	6	9	10
	VS9	20	20	60	5	40	55	13	15
	VS10	33.3	33.3	33.3	26	11	63	20	22
RTM + KW + PCA	VS1	80	10	10	46	50	4	26	30
	VS2	10	80	10	17	70	13	7	8
	VS3	10	10	80	13	17	70	7	8
	VS4	40	30	30	42	35	23	5	5
	VS5	30	40	30	68	16	16	25	27
	VS6	30	30	40	9	59	32	19	21
	VS7	60	20	20	14	68	18	32	38
	VS8	20	60	20	17	50	33	9	9
	VS9	20	20	60	15	21	64	3	4
	VS10	33.3	33.3	33.3	17	46	37	11	12

Overall, based on the estimates of MAE and RMSE (< 20% for the majority of VS mixtures), GLUE provided accurate predictions of the spatial source contributions in the study area. Equally successful applications of GLUE for source fingerprinting aeolian sands and atmospheric dust, in Australia and Iran respectively, have been reported by^14,21^. The GLUE framework can therefore return acceptable accurate estimates of source contributions in different environmental settings and for different types of target sediment.

## Study limitations

Any SSF study necessarily has inherent limitations and uncertainties. Available resources and in many cases, the nature of the landscape in question, inevitably serve to constrain field access and effort and sampling campaigns. In this study, a conventional confluence-based approach to estimating sub-basin spatial sediment source contributions^50,51^ was implemented using limited sampling of the spatial sources and downstream target sediment and the single campaign nature of these samples should be borne in mind when interpreting the results. The river bed sediment samples used to represent the spatial sources and the downstream target sediment were not age-dated^52–54^ and so it is not possible to confirm the time period represented by the source apportionment estimates. Although a combination of two range tests and tracer biplots was used to help evidence tracer conservatism, it remains widely reported that these approaches fail to attest to the complete absence of transformation during sediment mobilization and routing. With regards the constituent tracers selected in the final composite signatures used in our work, Ca, Mn and P have been reported as being susceptible to phase changes, whereas in comparison, Al, Ce, Eu, La and Sc have been reported as being far less susceptible to mobility between phases^55^. The composite signatures selected in this study are essentially statistical solutions generated by different combinations of tests and here it is informative to bear in mind that some studies have attempted to focus on knowledge-based pre-selection of tracers^56,57^. Finally, VS mixtures were used as a convenient and widely adopted^58,59^ means to assess the accuracy of the predicted spatial source proportions, since independent monitoring data for the sediment loads emitted from the tributary sub-catchments was not available. The lack of independent evidence to validate SSF results is common to almost all existing published studies, although a few exceptions exist^25,60^.

## Conclusions

The novel contribution of this study is quantification of the uncertainty and accuracy of the spatial source contributions from three sub-basins of a large arid catchment in southern Iran using SSF within a GLUE framework. Based on MAF and GOF, all four tracer models performed well, but GLUE with the final composite signature selected by the more traditional statistical procedures (TRT + KW + DFA and RTM + KW + DFA) for source discrimination performed slightly better than the two other models (GLUE with TRT + KW + PCA, and GLUE with RTM + KW + PCA). Our results for sediment source apportionment can help managers target spatial priorities for interventions to help decrease sediment loads and mitigate their negative effects in the Mehran study catchment. On the basis of our experience with this study, we recommend applying the SSF approach within a GLUE framework with the traditional statistical tests for quantifying source contributions. To confirm the wider applicability of our case study findings, our analyses herein should be replicated for estimating the sources of aeolian and fluvial sediments and the associated uncertainties and accuracies in catchments and landscapes located in different regions around the world experiencing severe soil erosion and sediment delivery by either water or wind.
